# Behavioral and Neural Dysregulation to Social Rewards and Links to Internalizing Symptoms in Adolescents

**DOI:** 10.3389/fnbeh.2019.00158

**Published:** 2019-07-23

**Authors:** Seh-Joo Kwon, Susannah L. Ivory, Ethan M. McCormick, Eva H. Telzer

**Affiliations:** ^1^Department of Psychology and Neuroscience, The University of North Carolina at Chapel Hill, Chapel Hill, NC, United States; ^2^Department of Psychology, Pennsylvania State University, State College, PA, United States

**Keywords:** adolescence, social reward, inhibitory failures, cognitive control, internalizing symptoms, connectivity, fMRI

## Abstract

Adolescence is a time of unique sensitivity to socially salient stimuli such as social rewards. This period overlaps with the onset of psychopathology such as internalizing and externalizing symptoms. In the current studies, we examined behavioral and neural patterns of dysregulation to social rewards and threats, and links to internalizing and externalizing symptoms in youths. In study 1, we used a social Go/NoGo cognitive control task using peer faces to test for age-related behavioral differences in inhibitory failures in adolescents (*N* = 53, M_age_ = 13.37 years), and adults (*N* = 51, M_age_ = 43.71 years). In study 2, an independent adolescent sample (*N* = 51, M_age_ = 13.98 years) completed a similar social Go/NoGo cognitive control task during fMRI. Results show that adolescents had greater inhibitory failures – as measured by false alarm rate – to both social reward and threat cues than adults, and more so to social reward than threat cues. Greater inhibitory failures to social reward than threat cues were associated with greater internalizing symptoms, but were not significantly related to externalizing symptoms. At the neural level, greater inhibitory failures to social reward than threat cues as well as greater internalizing symptoms were both associated with heightened amygdala-ventral striatum connectivity. Our findings indicate that subcortico-subcortical connectivity, which is deemed to occur chronologically earlier and thus necessary for subcortico-cortical circuits, may serve as an early biomarker for emotion dysregulation and a risk factor for internalizing symptoms.

## Introduction

Adolescence is a period of unique development characterized by a social reorientation in the brain (Nelson et al., 2005). That is, the adolescent brain undergoes neural plasticity and growth during the onset of puberty such that it becomes more sensitive to socially salient stimuli in the environment (Blakemore, 2008; Crone and Dahl, 2012; Pfeifer et al., 2013). During this neurobiological transformation, the adolescent brain shows greater sensitivity to social rewards as evidenced by heightened recruitment of limbic regions (e.g., amygdala, ventral striatum) in response to socially affective cues (Crone and Dahl, 2012; Galván, 2013). This social reorientation explains, in part, why peers become an increasingly powerful influence in adolescents’ lives, and why adolescents become more driven by socially appetitive cues such as social rewards (Galván, 2013; Smith et al., 2015; Foulkes and Blakemore, 2016). This bias toward social rewards may facilitate adolescents’ desire to seek and value peer acceptance and group membership more so than children and adults (Brown et al., 1986; McElhaney et al., 2008), guiding adolescents to adjust their motivations to match their social context, and needs (Crone and Dahl, 2012). While developmentally normative (e.g., Perino et al., 2016), this heightened orientation to peer acceptance and social rewards may lead to emotion dysregulation (Masten et al., 2009; Breiner et al., 2018), and place adolescents at risk for psychopathology (Nelson et al., 2005).

During the adolescent years, a social reorientation toward peers and gaining social acceptance coincides with a heightened risk for psychopathology including internalizing (e.g., depression and anxiety) and externalizing (e.g., impulsivity, aggression, and conduct problems) symptoms (e.g., Achenbach, 1966; Costello et al., 2011). Internalizing and externalizing symptoms involve affective dysregulation and compromised executive functioning (Kerestes et al., 2014; Mullin et al., 2018) such as poorer cognitive control (Snyder and Hankin, 2016), as measured by lower inhibitory control (Schulz et al., 2004; Vuontela et al., 2013), and altered reaction times during inhibitory failures (Albrecht et al., 2005; Ladouceur et al., 2006). This ultimately has lasting implications on adolescents’ lives (e.g., Fergusson and Woodward, 2002; Bongers et al., 2008). For instance, youths with internalizing and externalizing symptoms are more susceptible to experience internalizing disorders and substance use, respectively, in the future (e.g., Pine et al., 1998; Fergusson and Woodward, 2002; King et al., 2004). Youths with internalizing symptoms also experience social dysfunction such that those who perceive low acceptance tend to be more depressed (Zimmer–Gembeck et al., 2007) while those with externalizing symptoms have atypical socially rewarding experiences (Foulkes et al., 2014). Given the prevalence and enduring impact of internalizing and externalizing symptoms, it is therefore necessary to better understand neurodevelopmental risk factors in youths.

Emotional dys(regulation) is thought to underlie both internalizing and externalizing symptoms in adolescence and arises due to neural changes in the developing brain (e.g., Casey et al., 2019). While many neurodevelopmental models have been proposed to explain adolescents’ enhanced orientation toward social rewards and their subsequent inabilities to engage in effective regulation [e.g., dual systems model (Steinberg, 2010); imbalance model (Casey et al., 2008)], these models and much of the empirical work focuses on cortico-subcortical (e.g., prefrontal cortex-ventral striatum) connectivity. However, prior to the development of down-regulation via the prefrontal cortex, emotional development is marked by a hierarchical cascade of changes in functional connectivity patterns, whereby development of subcortico-subcortical connectivity (e.g., amygdala-ventral striatum connectivity) occurs before that of cortico-subcortical connectivity, and serves as a necessary precursor to more complex neural interactions (Casey et al., 2019).

To date, there has been a wealth of research on amygdala and ventral striatum activation in tandem, however, only a few have probed connectivity between the two subcortical regions in humans. Amygdala-VS connectivity plays a vital role in relevance detection (Ousdal et al., 2012), affective valuation (Everitt and Robbins, 1992), and incentive-based learning (Fareri et al., 2015), which may promote downstream motivated cognition, and behavior (Ousdal et al., 2012; Fareri et al., 2015). Longitudinal (Pfeifer et al., 2011) and cross-sectional (Heller et al., 2016) studies highlight developmental decreases in amygdala-VS connectivity from childhood to adulthood, suggesting that strengthened connectivity between these regions is a developmentally immature neural phenotype and may underlie difficulties in emotion regulation in adolescence. Indeed, greater amygdala-VS connectivity is associated with behavioral disinhibition to emotional cues (Heller et al., 2016), which may place youth at risk for psychopathology. While there indeed is a large body of literature on the links between alternations in amygdala and ventral striatum activation and internalizing and externalizing symptoms in adolescents, especially in a socially rewarding context (e.g., Scheres et al., 2007; Guyer et al., 2008; Monk et al., 2008; Davey et al., 2011; Telzer et al., 2014; Olino et al., 2015; Fareri and Tottenham, 2016), little to no research has probed how alterations in amygdala-VS connectivity relate to internalizing and externalizing symptoms (but see Roy et al., 2013). This calls for further investigation into how maladaptive processing of social rewards relate to subcortico-subcortical connectivity and internalizing and externalizing symptoms.

In the current studies, we sought to investigate the behavioral and neural correlates of disinhibition to socially affective cues (social rewards and social threats) and links to internalizing and externalizing symptoms in adolescents. In the current study, participants completed a social Go/NoGo task where “go” and “no-go” cues were superimposed onto social reward (e.g., happy peer face), social threat (e.g., angry peer face), or neutral (i.e., neutral peer face) images. Past studies have utilized similar Go/NoGo tasks to assess inhibitory failures operationalized by false alarm rates (i.e., pressing a button on no-go trials; e.g., Somerville et al., 2011; Perino et al., 2016). Positive (e.g., happy) and negative (e.g., angry) facial expressions serve as social reinforcers that induce approach/reward and avoidance/threat responses, which can alter the probability of enacting executive functions such as response latencies (e.g., Hare et al., 2005; Kohls et al., 2009). Thus, happy and angry faces are frequently used in fMRI research to elicit social reward and social threat processing, respectively (e.g., Gorno-Tempini et al., 2001; Hare et al., 2008; Somerville et al., 2011; Cremers et al., 2015). Moreover, social reward (happy faces) and social threat (angry faces) cues recruit amygdala-striatal circuitry (Pfeifer et al., 2011; Heller et al., 2016).

In study 1, adolescent and adult participants completed the social Go/NoGo task behaviorally to test for developmental differences. The task was developmentally congruent, such that adolescents viewed adolescent faces and adults viewed adult faces. The goal of study 1 was to ensure ecological validity of the task that utilizes peer faces by replicating prior behavioral findings that have shown that adolescents relative to children and adults make more false alarms in the presence of social reward cues (Somerville et al., 2011; Perino et al., 2016). Thus, we hypothesized that adolescents relative to adults would show greater behavioral disinhibition to social reward cues relative to social threat and neutral cues (Somerville et al., 2011; Perino et al., 2016).

In study 2, an independent sample of adolescents completed the social Go/NoGo task during an fMRI session. Prior developmental neuroimaging work has shown that adolescents show greater amygdala-VS connectivity relative to adults, and heightened connectivity is associated with greater behavioral disinhibition to emotional cues on a social Go/NoGo task (Heller et al., 2016). Thus, we hypothesized that greater disinhibition to social reward cues would be associated with greater amygdala-VS connectivity since heightened subcortical coupling is seen as developmentally immature (Casey et al., 2019).

Finally, we examined behavioral and neural links with internalizing and externalizing symptoms. At the behavioral level, we hypothesized that greater disinhibition to social reward cues relative to social threat cues would be associated with higher internalizing and externalizing symptoms. At the neural level, we hypothesized that stronger amygdala-VS connectivity to social rewards would be associated with greater internalizing and externalizing symptoms.

## Materials and Methods

### Participants

Participants consisted of a community sample recruited via flyers, listservs, and outreach at local events. We obtained informed consent/assent from all participants. The University’s Institutional Review Board approved all procedures and materials.

#### Study 1 (Behavioral)

Participants included 51 adults (M_age_ = 43.71 years, *SD* = 6.76 years, range = 27.49–55.91 years; 41 female; 31 White, 13 African American/Black, 2 Asian/Pacific Islander, 2 Latino/Hispanic, and 3 multiethnic) and 55 adolescents. Two adolescent participants were excluded from study 1 due to an inability to follow the task instructions, leaving a total of 53 adolescent participants (M_age_ = 13.37 years, *SD* = 0.61 years, range = 12.18–14.82 years; 27 female; 25 White, 14 African American/Black, 4 Asian/Pacific Islander, 2 Latino/Hispanic, and 8 multiethnic; Maternal education: 1 some high school, 3 high school degree, 12 some college, 21 college degree, 1 some medical, law, or graduate school, 14 medical, law, or graduate school degree, 1 missing).

#### Study 2 (fMRI)

Participants included an independent sample of 59 adolescents. 7 participants were excluded from analyses because they could not complete the task properly (e.g., technical problems, misunderstanding of task) and 1 participant was excluded because of excessive motion during the scan. In total, 51 participants were included in the present analyses (M_age_ = 13.98 years, *SD* = 1.24 years, range = 12.03–15.94 years; 25 female; 32 White, 9 African American/Black, 1 Asian/Pacific Islander, 1 Latino/Hispanic, and 8 multiethnic; Maternal education: 4 some high school, 4 high school degree, 2 trade or vocational schools, 8 some college, 19 college degree, 3 some medical, law, or graduate school, 11 medical, law, or graduate school degree).

### Social Go-Nogo Task

#### Study 1 (Behavioral)

**FIGURE 1:**
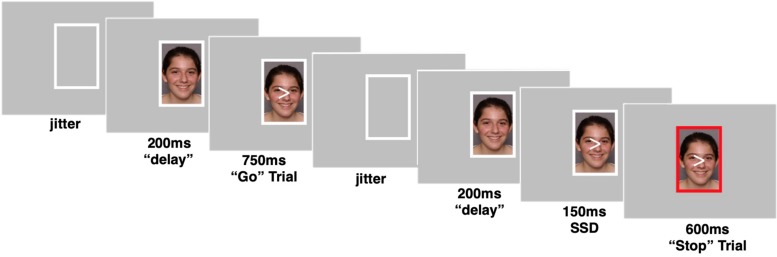
Example trials of the social stop signal task. Go trials when frame enclosing peer’s photo remains white and No-go trials when frame enclosing peer’s photo turns red. Pictures were taken from a publicly available dataset (Egger et al., 2011). Parent permission and actor assent were obtained by a contractual arrangement so that pictures are publicly available for researchers and can be reproduced in scientific dissemination.

Participants completed a behavioral inhibition task, during which they were instructed to inhibit a motor response in the presence of happy, angry, and neutral faces (Figure 1). Participants viewed a sequence of arrows (“<” or “>”) superimposed on top of pictures of faces enclosed within a white rectangular frame. Participants were instructed to press a button with their right or left pointer finger depending on the direction of the arrow. No instructions were given regarding the faces (e.g., participants were not told to attend to the faces in any way). In some trials, the white frame would turn red and participants were instructed to withhold their response if the frame turned red. The faces in the photos were age-matched such that the adolescents viewed photos of adolescents [drawn from the NIMH Child Emotional Faces Picture Set (NIMH-ChEFS); Egger et al., 2011] and adult participants viewed adult faces (drawn from the NimStim; Tottenham et al., 2009). Faces were of diverse races and ethnicities. Each photo displayed one of three emotional facial expressions: happy, angry, or neutral. The same faces (with different facial expressions) were displayed in all 3 conditions.

The task consisted of 207 trials in total, which were divided by emotional facial expression into 3 blocks of 69 trials each. Within each block, two thirds of the trials (46) were “go” trials, where the correct response was to press a button. One third of the trials (23) were “no-go” trials, where the correct response was to withhold a button press. The direction of the arrow (“<” or “>”) was assigned randomly to each trial. During a go trial, the photo was first presented for 200 ms within the white frame, then an arrow appeared superimposed on top of the photo for 750 ms. Next, the photo and arrow disappeared, leaving only the white frame for a jittered intertrial period. During a no-go trial, the photo was presented for 200 ms within the white frame, then the arrow appeared superimposed on top of the photo for 150 ms, while still enclosed within the white frame. Next, the frame surrounding the photo and arrow turned red for 600 ms. Then the photo and arrow disappeared, and the frame returned to its original white color for the jittered intertrial period.

#### Study 2 (fMRI)

The task used in study 2 was extremely similar to that described above for study 1 with minor updates to optimize the task for fMRI use. The number of trials was increased to a total of 333 trials with 111 trials per emotion block. The ratio of go to no-go trials was kept at two thirds go (74) trials and one third no-go (37) trials within each block. Additionally, the task was updated so that the task difficulty would adapt to the individual’s performance, ensuring the task is similarly, difficult across participants. Specifically, the amount of time before the frame turned red (referred to here as the “Stop Signal Duration” or SSD) on no-go trials adapted to the participants’ performance. The SSD was variable and was determined by the participant’s performance on the task. If a participant successfully withheld a button press on a no-go trial, then the SSD for the next no-go trial would increase by 50 ms, making the task more difficult. Conversely, if a participant failed to withhold their button press on a no-go trial, the SSD for their next no-go trial would decrease by 50 ms. The initial SSD was set to 150 ms, and bounded at 50 ms (minimum) and 350 ms (maximum). A go trial in the task followed the same pattern and timing as described in study 1. A no-go trial in study 2 followed this sequence: the photo was presented within the white frame for 200 ms. The arrow then appeared superimposed on the top of the photo for a variable SSD, after which the frame turned red. The frame remained red for the period of time necessary for the total amount of time the arrow was displayed to equal 750 ms. For example, if the SSD was 250 ms, the red frame was displayed for 500 ms. Finally, the arrow and photo disappeared for a jittered intertrial period.

### Self-Report Measures

To measure internalizing and externalizing symptoms, adolescents in study 2 completed the Strengths and Difficulties Questionnaire (SDQ; Goodman, 1997). Internalizing symptoms were measured using the Emotional and Peer Problems subscales and externalizing symptoms were measured using the Behavioral and Hyperactivity subscales. For each measure, the combination of the two subscales created a second-order factor that measures broad internalizing or externalizing symptoms, especially for low-risk, non-clinical youth samples (Goodman et al., 2010). Adolescents reported the extent to which the 10 items of internalizing symptoms (e.g., “I am often unhappy, down-hearted or tearful”) and 10 items of externalizing symptoms (e.g., “I am often restless, overactive, cannot stay still for long”) were true of them. Participants use a 3-point Likert scale (0 = *Not True* to 2 = *Certainly True*). Scores were calculated as the sum of the 10 items for each measure (α = 0.64 for internalizing, α = 0.75 for externalizing). Mean scores in the current sample were 5.86 (SE = 0.47; median = 5; range = 1–14) for internalizing symptoms and 6.04 (SE = 0.54; median = 6; range = 0–14) for externalizing symptoms.

### fMRI Data Acquisition

Imaging data were collected using a 3 Tesla Siemens Magnetom Trio MRI scanner. The task consisted of T2^*^-weighted echoplanar images (EPI; 300 volumes; slice thickness = 3 mm; 38 slices; TR = 2 s; TE = 25 ms; matrix = 92 × 92; FOV = 230 mm; voxel size = 2.5 mm^3^ × 2.5 mm^3^ × 3 mm^3^). Structural scans, including a T1^*^ magnetization-prepared rapid-acquisition gradient echo (MPRAGE; 192 slices; TR = 1.9 s; TE = 2.32 ms; FOV = 230 mm; matrix = 256 × 256; sagittal acquisition plane; slice thickness = 0.9 mm) and a T2^*^-weighted, matched-bandwidth (MBW), high resolution anatomical scan (38 slices; TR = 4 s; TE = 64 ms; FOV = 230 mm; matrix = 192 × 192; slice thickness = 3 mm) were also acquired. To maximize brain coverage and reduce drop-out in orbital and temporal regions, MBW and EPI images were acquired at an oblique axial orientation.

#### fMRI Data Preprocessing and Analysis

Preprocessing steps, utilizing FSL FMRIBs Software Library (FSL v6.0^1^), included the following: skull stripping of all images using BET; slice-to-slice motion correction of EPI images using MCFLIRT; sequential co-registration of EPI images to standard stereotactic space defined by the Montreal Neurological Institute (MNI) and the International Consortium for Brain Mapping through the MBW and MPRAGE images using FLIRT; application of a 128 s high-pass temporal filter to remove low frequency drift within the time-series; and spatial smoothing with a 6 mm Gaussian kernel, full-width-at-half maximum. Individual-level independent component analysis (ICA) using MELODIC was applied and combined with an automated component classifier (Tohka et al., 2008; Neyman-Pearson threshold = 0.3) in order to remove artifact signal (e.g., physiological noise, motion) from the functional data. Quality check during preprocessing and analyses ensured adequate signal coverage in our sample.

The task was modeled using an event-related design within the Statistical Parametric Mapping software package (SPM8; Wellcome Department of Cognitive Neurology, Institute of Neurology, London, United Kingdom). Each event was modeled using the onset of the stimulus and a duration equal to the participants’ response time (or 750 ms on trials where participants did not respond). Individual fixed-effects models were created for each participant using the general linear model in SPM with regressors for conditions of interest: trials during each emotion block (e.g., neutral, happy, and angry). Consistent with prior work (Perino et al., 2016; Rogers et al., in press), all trials were modeled within a single regressor for a given block of the task, regardless of outcome, in order to capture the neural correlates involved in processing social rewards and social threats. Volumes containing motion in excess of 2 mm slice-to-slice were modeled in a separate junk regressor. However, if the number of volumes that exceeded the threshold was greater than 10% of the total number of trials, then the participant was excluded from the analyses. Jittered inter-trial periods (e.g., fixation) were not explicitly modeled and therefore serve as the implicit baseline for task conditions.

We conducted psychophysiological interaction (PPI) analyses using a generalized form of context-dependent PPI from the automated generalized PPI (gPPI) toolbox in SPM (McLaren et al., 2012). In order to examine amygdala-striatum functional connectivity, we used the bilateral ventral striatum as our seed region, which was defined structurally from WFU pickatlas (Maldjian et al., 2003) using the AAL atlas (Tzourio-Mazoyer et al., 2002) with the following restrictions: −12 < *x* < 12, 4 < *y* < 8, −12 < *z* < 0. Time series were extracted from the VS seed region and served as the physiological variable. Each block of trials was then convolved with the canonical HRF to create the psychological regressor. In the final step, the physiological and psychological variables were multiplied in order to create the PPI term. This interaction term was then used to identify regions that covary with the ventral striatum seed region in a task-dependent manner. As such, each participant’s individual gPPI model included a deconvolved BOLD signal alongside the psychological and interaction term for each event type.

Random effects, group-level analyses were run using GLMFlex^2^. GLMFlex offers several advantages, including removing outliers and sudden activation changes in brain, corrects for variance-covariance inequality, partitions error terms, and analyzes all voxels containing data. Group-level analyses were performed by entering the number of false alarms committed by participants and self-reported internalizing/externalizing symptoms as continuous covariates in a series of whole-brain regressions, first testing for associations with neural activation followed by our key analysis on analyses on amygdala-VS functional connectivity.

Monte Carlo simulations were used to compute a cluster corrected threshold using the updated (April, 2016) 3dFWHMx and 3dClustSim programs from the AFNI software package (Ward, 2000) and the group-level brain mask for the analyses of interest. Simulations resulted in a voxel-wise threshold of *p* < 0.005 and a minimum cluster size ranging between 117 and 380 voxels for the whole-brain, corresponding to *p* < 0.05, family-wise error (FWE) corrected. For our *a priori* analyses focused on amygdala-VS connectivity, we utilized a small-volume correction, computing a cluster corrected threshold within a structurally defined amygdala mask from the AAL atlas. Simulations resulted in a voxel-wise threshold of *p* < 0.005 and a minimum cluster size of 3 voxels within the amygdala, corresponding to *p* < 0.05 small volume corrected. All reported results are available on NeuroVault^3^ (Gorgolewski et al., 2015).

## Results

### Behavioral Results

#### False Alarm Rates to Social Rewards and Threats, Study 1

**FIGURE 2:**
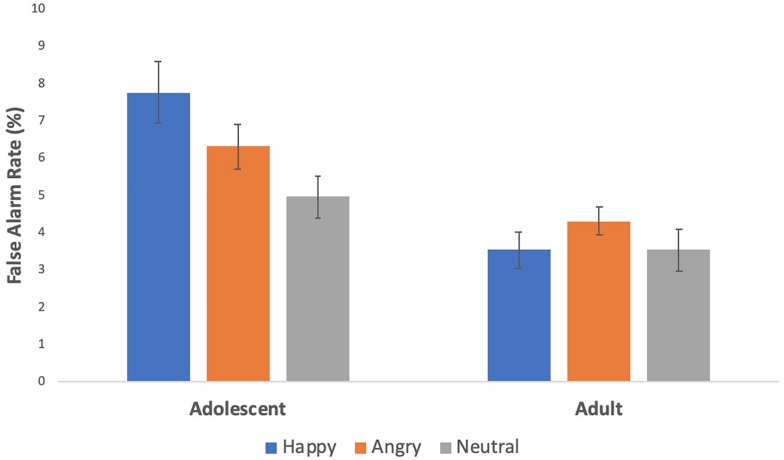
Behavioral effects on the social stop signal task. Adolescents had greater inhibitory failures to social reward and threat cues than to neutral cues, and more so to social reward than threat cues.

To test for age differences in false alarm rates across happy, angry, and neutral blocks, we conducted a repeated measures analysis of variance with one within subject variable (condition: happy, angry, and neutral) and one between subject variable (age group: adolescents, adults). Results revealed a significant main effect of condition, *F*(2,204) = 6.43, *p* = 0.002, η^2^ = 0.059 and group, *F*(1,102) = 13.47, *p* < 0.0001, η^2^ = 0.117 which was qualified by an age x condition interaction, *F*(2,204) = 6.60, *p* = 0.002, η^2^ = 0.061. To probe this interaction, we conducted paired samples *t*-tests within each age group. As shown in Figure 2, adolescents showed more false alarms to happy [*t*(52) = 4.37, *p* < 0.0001, *d* = 0.54] and angry faces [*t*(52) = 2.63, *p* = 0.01, *d* = 0.32] than neutral faces, and more false alarms to happy than angry faces [*t*(52) = 2.39, *p* = 0.02, *d* = 0.27]. Adults did not show any significant differences across conditions. Next, we conducted independent samples *t*-tests across the 2 age groups. Adolescents showed more false alarms than adults to happy [*t*(102) = 4.31, *p* < 0.0001] and angry faces [*t*(102) = 2.78, *p* = 0.007] but did not differ significantly to neutral faces [*t*(102) = 1.8, *p* = 0.074].

#### False Alarm Rates to Social Rewards and Threats, Study 2

We conducted a repeated measures analysis of variance with one within subject variable (condition: happy, angry, and neutral) to examine differences in false alarm rates across conditions in the adolescent sample. We found a significant effect of condition, *F*(2,116) = 3.4, *p* = 0.036, η^2^ = 0.056. *Post hoc*, paired samples *t*-tests corroborated the findings from study 1 and our prior work (Perino et al., 2016), such that adolescents made significantly more false alarms to happy (*M* = 19.29%, SE = 0.91%) compared to angry faces (*M* = 17.46%, SE = 0.91%; [*t*(58) = 2.71, *p* = 0.009, *d* = 0.26)]. However, false alarm rates to happy and angry faces did not differ from neutral faces. The fMRI version of the task includes the SSD, which adapts to participants’ behavior ensuring participants perform at a more fixed rate across the task, and so false alarm differences are harder to identify. It is thus not surprising that our behavioral effects are weaker, but it is nonetheless impressive that they still emerged in the expected direction.

#### False Alarm Rates to Social Rewards and Threats and Links to Internalizing and Externalizing Symptoms

To understand links between disinhibition to social rewards and psychopathology, we examined the relationship between disinhibition to social rewards relative to social threats and internalizing symptoms in adolescents. We calculated a difference score for false alarm rates by subtracting false alarms rates to angry faces from happy faces, where higher scores indicate adolescents make more false alarms to social rewards. Adolescents who had greater false alarm rates to happy relative to angry faces reported greater internalizing symptoms [*r*(50) = 0.33, *p < 0*.05]. There was no significant correlation between false alarm rate to happy relative to angry faces and externalizing symptoms [*r*(50) = 0.12, *p =* 0.39].

### fMRI Results

#### Main Effects of Social Rewards > Social Threats

Given the heightened false alarm rates to happy relative to angry faces, we focused our analyses on this specific contrast. We first conducted a whole-brain *t*-test that compared happy and angry faces. Next, we investigated functional connectivity for this contrast. Results are shown in Table 1.

**TABLE 1:** Brain activation patterns for neural activation and functional connectivity.

***Anatomical region***	***x***	***y***	***z***	***t***	***k***
*Social reward > social threat*					
*PPI (VS seed): Social reward > social threat*					
L Middle frontal gyrus	−26	14	46	−4.18	271
L Medial cingulate cortex	−2	−4	40	−3.26	226
Supplementary motor area	10	0	64	−4.09	312
L Inferior parietal lobule	−52	−38	44	−3.52	232
*False alarm rate regressed on social reward > social threat*					
R Amygdala	26	8	−22	−3.51	83
L Amygdala	−14	0	−18	−3.48	63
*PPI (VS seed): False alarm rate regressed on social reward > social threat*					
L Amygdala	−24	−4	−14	3.16	34
*PPI (VS seed): False alarm rate regressed on social reward > neutral*					
L Amygdala	−22	−4	−10	3.43	22
Dorsomedial prefrontal cortex	−10	66	24	4.70	367
Superior temporal sulcus	−60	−24	0	3.76	150
L Cerebelum	−18	−76	−36	3.75	133
*PPI (VS seed): False alarm rate regressed on social treat > neutral*					
*Internalizing regressed on social reward > social threat*					
*PPI (VS seed): Internalizing regressed on social reward > social threat*					
L Amygdala	−24	−6	−12	2.86	7
L Amygdala	−16	0	−16	3.93	76
L Interior frontal gyrus (p. Orbitalis)	−26	26	−12	5.16	179
R Postcentral gyrus	32	−42	70	4.40	669
L Postcentral gyrus	−38	−38	64	3.96	327
L Middle frontal gyrus	−28	−2	66	4.13	374
L Anterior insula	−44	12	−16	4.07	120
R Posterior insula	28	−18	0	3.86	369
R Supramarginal gyrus	64	−24	28	4.00	183
*PPI (VS seed): Internalizing regressed on social reward > neutral*					
L Amygdala	−22	−2	−14	3.77	68
R Cuneus	20	−84	34	5.10	1208
L Anterior insula	−36	0	10	4.49	356
Supplementary motor area	0	−16	68	4.41	117
Supplementary motor area	6	−8	58	3.68	269
R Caudate	8	2	8	3.88	323
*PPI (VS seed): Internalizing regressed on social treat > neutral*					
*Externalizing regressed on social reward > social threat*					
Posterior superior temporal sulcus	−64	−38	−4	−3.89	465
*PPI (VS seed): Externalizing regressed on social reward > social threat*					
R Angular gyrus	56	−62	36	3.79	136
L Temporoparietal junction	−46	−60	26	3.7	128

*PPI refers to psychophysiological interaction. L and R refer to left and right hemispheres, respectively. *k* refers to the number of voxels within that cluster, *t* refers to peak activation level within that cluster, and *x*, *y*, *z* refer to MNI coordinates. The amygdala was small-volume corrected. All other regions were based on whole-brain mask (range = 117–380 voxels). All regions are significant at *p* < 0.005.*

#### Neural Correlates of False Alarm Rate to Social Rewards and Threats

Next, we examined how behavioral disinhibition relates to neural activation and amygdala-VS connectivity. Using the same behavioral metric as described above, we regressed the difference in false alarm rates (happy-angry) onto neural activation and neural connectivity for the contrast happy-angry. For neural activation, we found a bilateral amygdala cluster, such that adolescents with greater false alarms to social reward relatives to threat show less activation in bilateral amygdala to social rewards (see Table 1).

**FIGURE 3:**
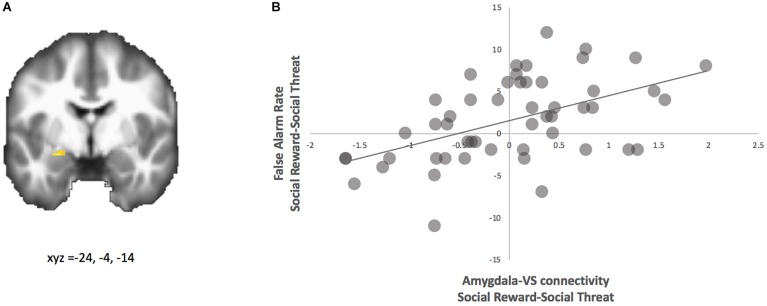
PPI analysis with ventral striatum seed. **(A)** Adolescents with greater false alarm rate to social reward-social threat trials showed greater functional connectivity between the ventral striatum and amygdala (highlighted; MNI coordinates: x, y, z = –24, –4, –14) for social reward > social threat. **(B)** Parameter estimates of connectivity strength were extracted for descriptive purposes and plotted with false alarm rate.

For neural connectivity, with the ventral striatum as the seed region, PPI analyses yielded coupling with the left amygdala that correlated with greater false alarm rates to happy faces (see Figure 3A and Table 1). For descriptive purposes, we extracted parameter estimates of functional connectivity. As shown in Figure 3B, adolescents who made more false alarms to happy relative to angry faces exhibited greater amygdala-VS connectivity to happy relative to angry faces. To further probe this effect, we examined how differences in false alarm rates are associated with neural connectivity to happy and angry faces separately (happy-neutral and angry-neutral). Using the ventral striatum as the seed region, PPI analyses demonstrated greater coupling with the left amygdala for happy relative to neutral cues that correlated with greater false alarm rates to happy faces. No significant correlation was found for angry relative to neutral cues (see Table 1). These findings suggest that failed inhibition to social rewards relative to threats may be facilitated amygdala-VS connectivity specifically to social reward cues.

#### Links to Internalizing and Externalizing Symptoms

**FIGURE 4:**
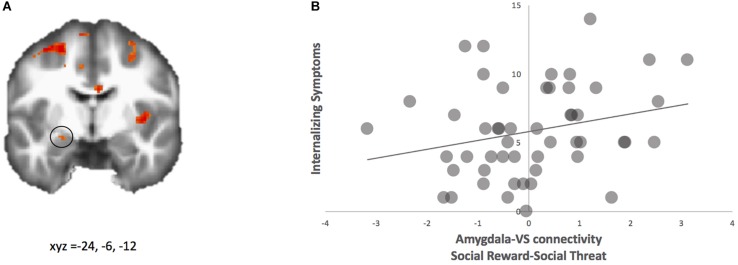
PPI analysis with ventral striatum as seed. **(A)** Adolescents with higher internalizing symptoms showed greater functional connectivity between ventral striatum and amygdala (highlighted; MNI coordinates: x, y, z = –24, –6, –12) for social reward > social threat. **(B)** Parameter estimates of connectivity strength were extracted for descriptive purposes and plotted with internalizing symptom scores.

We examined how amygdala-VS connectivity is associated with internalizing and externalizing symptoms. First, we regressed internalizing symptoms onto neural activation and neural connectivity for the contrast happy-angry. For neural activation, no significant clusters were observed. For neural connectivity, using the ventral striatum as the seed region, the PPI analyses yielded coupling with the left amygdala that correlated with internalizing symptoms (see Figure 4A and Table 1). This region is nearly identical to that found above for the connectivity analyses regressed with false alarm rate. For descriptive purposes, we extracted parameter estimates of functional connectivity. As shown in Figure 4B, adolescents who showed greater connectivity to happy relative to angry faces reported greater internalizing symptoms. To further probe this effect, we examined how neural connectivity to happy and angry faces separately (happy-neutral and angry-neutral) relate to internalizing symptoms. Using the ventral striatum as the seed region, PPI analyses showed connectivity with the left amygdala for happy relative to neutral cues that correlated with internalizing symptoms. No significant correlation was found for angry relative to neutral cues.

Next, we regressed externalizing symptoms onto neural activation and neural connectivity for the contrast happy-angry. Results for neural activation are shown in Table 1. Furthermore, results for neural connectivity using the ventral striatum as the seed region did not yield coupling with the amygdala that correlated with externalizing symptoms (see Table 1). We therefore did not continue to analyze whether happy and angry faces separately (happy-neutral and angry-neutral) relate to externalizing symptoms.

## Discussion

Adolescents demonstrate a rise in sensitivity to socially affective cues such as social rewards (Guyer et al., 2012), which overlaps with a heightened risk for psychopathology such as internalizing and externalizing symptoms (e.g., Costello et al., 2011). The aim of the current study was to examine neural and behavioral dysregulation to social rewards and links to internalizing and externalizing symptoms in youths. Our results suggest that greater behavioral disinhibition to social reward cues (i.e., happy peer faces) than to social threat cues (i.e., angry peer faces) is associated with heightened amygdala-VS connectivity in adolescents. Moreover, greater internalizing, but not externalizing, symptoms were associated with greater behavioral disinhibition to social rewards as well as amygdala-VS connectivity. Together, these findings indicate that greater disinhibition to social rewards may render adolescents at greater risk for internalizing symptoms due to their shared amygdala-VS connectivity to social rewards relative to threats.

Behaviorally, adolescents showed greater inhibitory failures in response to socially affective cues – both social reward and social threat cues – than to neutral cues, and even more so to reward than to threat cues. Moreover, there were age-related differences such that adolescents had greater inhibitory failures to socially affective cues than adults who performed relatively uniformly across these various cues. These behavioral findings align with previous research in that adolescents are particularly sensitive to socially appetitive cues such as social rewards (Somerville et al., 2011; Perino et al., 2016), and extend this work by using peers’ faces. Given the intensified reward sensitivity in adolescents (Galván, 2013), it is plausible that adolescents demonstrate a stronger bias toward positive than negative cues, resulting in behavioral dysregulation in the presence of social rewards. Socially salient stimuli and information are especially relevant to adolescents, and ultimately shape their behavior (Nelson et al., 2005). Paying closer attention to social information at the cost of inhibitory failures may not necessarily be unfavorable to adolescents. Adolescence is a developmental period of social reformation where there are major changes in one’s social network such as forming new, meaningful social connections. For instance, adolescents start to enter romantic relationships (Furman and Wehner, 1997), and non-parental figures or non-family members (e.g., teacher, coach) begin to serve pivotal roles (Wang et al., 2013). Therefore, greater cognitive allocation to social information, such as positive social cues, may facilitate stronger social relationships in youths.

Hyper-sensitivity to socio-affective cues may come at a cost and ultimately place youth at risk for psychopathology. Indeed, greater inhibitory failures to social reward relative to social threat cues were associated with greater internalizing symptoms. Youths with greater internalizing symptoms, but not externalizing symptoms, tend to have better emotion comprehension such as understanding of others’ emotions (Göbel et al., 2016). In a social context, adolescents with internalizing symptoms have better identification of happy than angry facial cues (Vanhalst et al., 2017) and have faster reaction times to happy than angry and fearful facial cues in Go/NoGo tasks (Stoycos et al., 2017). This may imply that these youths at risk are particularly more sensitive to socially rewarding stimuli, which corroborate our finding of the relationship between behavioral disinhibition and internalizing symptoms.

Our study did not find a significant link between disinhibition and externalizing symptoms. Previous research on disinhibition and externalizing symptoms in adolescents demonstrates conflicting results. That is, while some research has shown that youth with externalizing symptoms make more false alarms on Go/NoGo tasks (Schulz et al., 2004; Bezdjian et al., 2009), others have found that there is no relationship between externalizing symptoms such as impulsivity and false alarms on Go/NoGo tasks (Brown et al., 2015; Sánchez-Kuhn et al., 2017). To our knowledge, this is the first study to examine the link between externalizing symptoms and behavioral disinhibition using salient peer faces in adolescents. It is possible that adolescents are just as impulsive to socially rewarding cues as they are to socially threatening cues. In other words, adolescents with symptoms of externalizing may be equally impulsive toward emotionally driven cues. However, given inconsistencies in results, further research is needed to better understand disinhibition in youths with symptoms of externalizing within a social context.

At the neural level, we found that adolescents who showed greater disinhibition to social reward cues demonstrated heightened connectivity between the amygdala and ventral striatum. Developmentally, connectivity between the two regions decreases from late childhood to early adolescence (Pfeifer et al., 2011), and continues to decrease in connectivity strength into early adulthood (Heller et al., 2016). Importantly, our findings corroborate a prior study such that adolescents who showed greater behavioral disinhibition to socio-emotional cues demonstrated heightened amygdala-VS connectivity (Heller et al., 2016). Greater connectivity between the amygdala and VS is thought to be a developmentally immature neural phenotype that emerges prior to the development of more mature top-down cortico-subcortical connectivity (Casey et al., 2019). This hierarchical cascade of changes in connectivity patterns (i.e., from subcortico-subcortical connectivity in early adolescence to cortico-subcortical connectivity in late adolescence to cortico-cortical connectivity in adulthood) is proposed to be necessary for emotional brain development (Casey et al., 2019). Together, our findings suggest that amygdala-VS connectivity, particularly in the context of social rewards, may represent a neural marker of emotion regulation difficulties.

Moreover, greater amygdala-VS connectivity was associated with greater internalizing but not externalizing symptoms. This coupling may underline an “unchecked” subcortical system that is characteristic of behavioral dysregulation to social rewards and compromised psychological well-being. While prior studies have examined the relationship between behavioral dysregulation and internalizing symptoms, which underscores connectivity between the cognitive control and affective hubs (e.g., Hare et al., 2008; Stoycos et al., 2017), our findings indicate that subcortico-subcortical connectivity, which is deemed to occur chronologically earlier and thus necessary for subcortico-cortical circuits (Casey et al., 2019), and may serve as an early biomarker for emotion dysregulation and a risk factor for internalizing symptoms. Putting these studies together, it can be reconciled that social context and neurobiology are key contributors to internalizing symptoms in adolescents.

There are several limitations to our study. First, we only had fMRI data for adolescents and therefore do not know whether these neural patterns are age-specific. Future studies should consider incorporating children and adult comparison groups or utilize longitudinal methods to see how behavioral differences map onto neural differences across development. Second, we used a community sample of adolescents with self-reported internalizing symptoms. Given that these adolescents were not clinically diagnosed, our findings cannot be extended to the community of youths with clinically relevant mood disorders. Nonetheless, we assessed internalizing symptoms in a community sample, suggesting that our findings may be more applicable to adolescents who are classified as healthy, but are not clinically diagnosed. Last, the Emotional and Peer Problems subscale of SDQ cannot be separated into depression and anxiety symptoms, and thus the two cannot be examined in tandem. However, it may be parsimonious to create a composite of internalizing symptoms given that depressive and anxiety symptoms tend to load on a higher-order internalizing symptoms factor. Future research should utilize longitudinal methods to better unpack the cascade of developmental processes that occur at the level of brain connectivity, behavioral disinhibition, and the onset of psychopathology.

In conclusion, the current study corroborates and extends previous work to better understand the contextual effects of disinhibition to social rewards on adolescent well-being. Our findings suggest that greater behavioral disinhibition to social rewards are associated with stronger amygdala-VS connectivity, where amygdala and ventral striatum are classified as “hot” affective nodes. Greater behavioral disinhibition and stronger amygdala-VS connectivity to social rewards are correlated with heightened internalizing symptoms, but not externalizing symptoms. Therefore, a greater orientation to social rewards may have implications for youth’s mental health such as depression and anxiety. These behavioral effects were also age-specific to adolescents, thereby confirming that socially salient contexts such as social rewards are especially powerful to youths’ motivations, behaviors, and psychological health.

## Data Availability

The datasets generated for this study are available on request to the corresponding author.

## Ethics Statement

This study was carried out in accordance with the recommendations of the Institutional Review Board at the University of Illinois with written informed consent/assent from all subjects. All subjects gave written informed consent in accordance with the Declaration of Helsinki.

## Author Contributions

ET and EM designed the research. SI and EM collected the data. SI and ET analyzed the data. All authors wrote the manuscript.

## Conflict of Interest Statement

The authors declare that the research was conducted in the absence of any commercial or financial relationships that could be construed as a potential conflict of interest.

## Footnotes

1 https://fsl.fmrib.ox.ac.uk/fsl/

2 http://mrtools.mgh.harvard.edu/index.php/GLM_Flex

3 https://neurovault.org/collections/5338/
